# Comparative study of sequential intravesical chemotherapy using gemcitabine and mitomycin C with mitomycin C alone for non-muscle invasive bladder carcinoma- a randomized trial

**DOI:** 10.1186/2051-1426-3-S2-P136

**Published:** 2015-11-04

**Authors:** Kumar Jayant, Santosh Kumar, Shrawan kumar Singh, Swati Agrawal, Rajendra Agrawal

**Affiliations:** 1 SHMRC/PGIMER, Kota, India

## Introduction

This study was conducted to investigate the ablative efficacy and safety of sequential intravesical gemcitabine and mitomycin C with mitomycin C alone in refractory non-muscle invasive bladder cancer (NMIBC).

## Methods

A total of 219 patients with refractory NMIBC were prospectively enrolled at tertiary academic center over period of Feb 2009-Jan 2012 & followed for next 3 yrs. They were randomly assigned to either of treatment arms: Gentamycin & mitomycin C (group A) or mitomycin C (group B). All patients underwent a 6-week induction regimen followed by a monthly maintenance regimen for one year if they responded to the induction course.

## Results

In Group A 98 of 102 & in group B 94 of 96 patients completed the therapy and were evaluated for response while 6 patient left the therapy in between. The therapy was well tolerated in the rest of patients. In group‘A’ i.e Gentamycin & mitomycin C a total, 82 patients (83.67%) exhibited a complete response to intravesical therapy. In 12.2% (12) patients had biopsy proven recurrence (22±6.16 months). In group ‘B’ (mitomycin C), 63 (65.60%) patients exhibited a complete response to intravesical therapy, 22 patients (22.9%) showed a partial response. During follow-up, 16 patients (25.3%) developed recurrence within this period. (14.5 +/- 8.26 months).

## Conclusions

Chemoresection with sequential intravesical gemcitabine and mitomycin C administration may be a viable option for BCG refractory non-muscle invasive bladder cancer (NMIBC).

**Figure 1:**
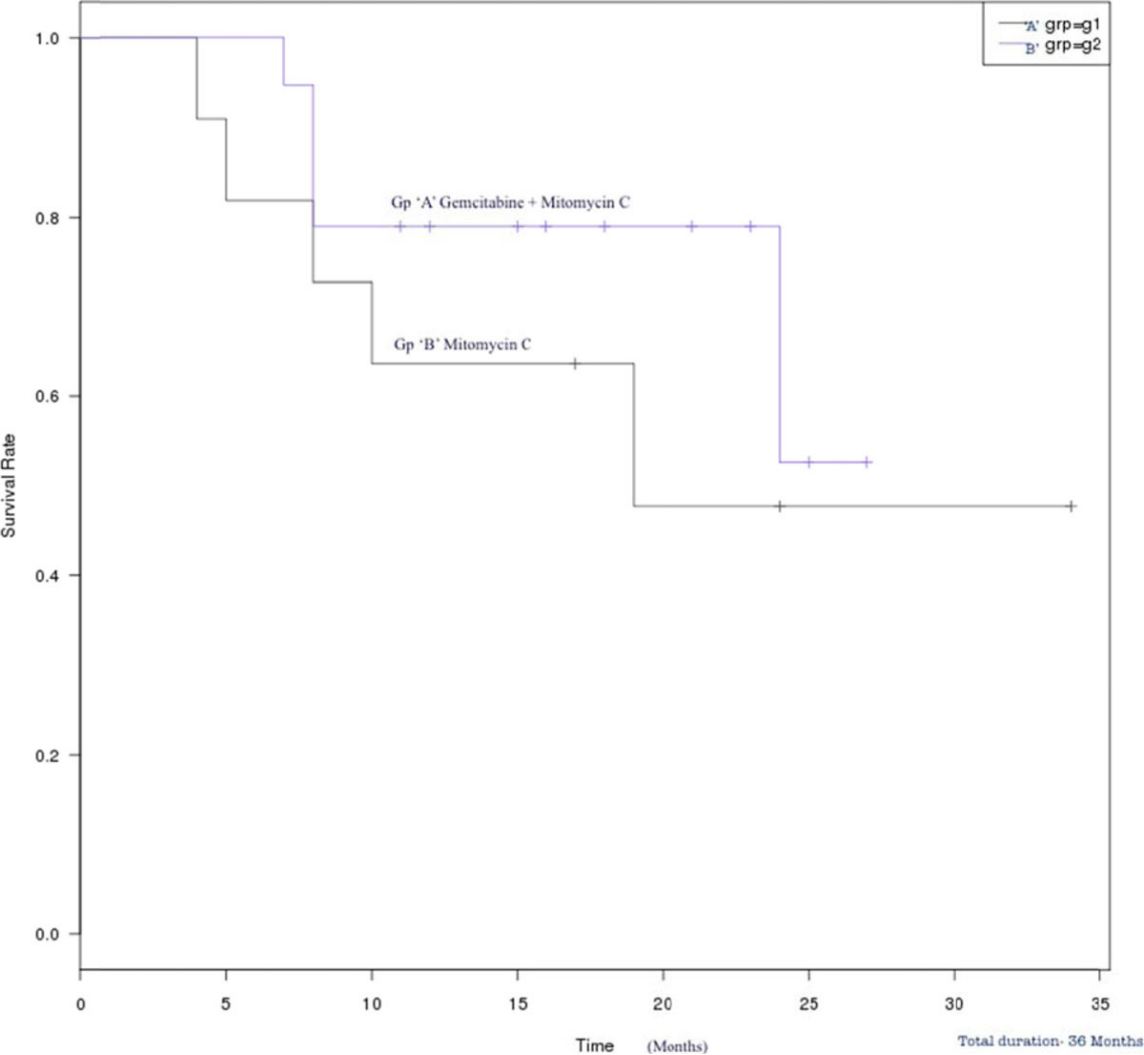


**Table 1:** Detail parameters of both groups.

Parameters	Group A	Group B	P value
	(MMC+Gemcitabine)	(MMC)	
Total patients	102	96	NS+
Male	81	78	NS+
Female	21	19	NS+
Age	65.7±9.8 yrs	63.9±8.9 yrs	NS+
Mean Duration of follow up	36 months	36 months	
Mean size of tumor <2cm	63	58	NS+
Mean sixe of tume >2 cm	39	38	NS+
Stage Ta	60	58	NS+
Stage T1	42	38	NS+
Grade 1	19	17	NS+
Grade 2	63	61	NS+
Grade 3	20	18	NS+
Previous treatment with BCG	All	All	
Aim of complete response	100%	100%	
Actual complete response	83.67% (82)	65.6% (63)	0.001*
Recurrence %	12.2% (12)	25.3% (16)	0.001*
Recurrence	22±6.16 months	14.5±8.26 months	0.001*
Recurrence range in months	8-36 months	4-14 months	

Values are presented as mean (+/- standard devitation)

Group A: Gemcitabine & Mitomycin (MMC+Gentamycin)

Group B: Mitomycin C(MMS)

*Statistical significance was analyzed by student t-test

+Statistical significance was analyzed by the chi-square test.

**Table 2:** Details of adverse effects of therapy in both groups.

Parameters	Group A	Group B	P value
	Gemcitabine + MMC	Mitomycin C(MMC)	
Dysuria	11.7% (12)	7.2% (7)	NS+
Suprapubic pain	11.7% (12)	9.3% (9)	NS+
Hematuria	3.9% (4)	3.1% (3)	NS+
Chemical Cystitis	7.8% (8)	6.25% (6)	NS+
Local reaction	6.8% (7)	4.16% (4)	NS+
Skin reaction	5.8% (6)	4.16% (4)	NS+

Group A: Gemcitabine & Mitomycin (MMC+Gentamycin)

Group B: Mitomycin C(MMS)

*Statistical significance was analyzed by student t-test

+Statistical significance was analyzed by the chi-square test.

**Figure 2:**
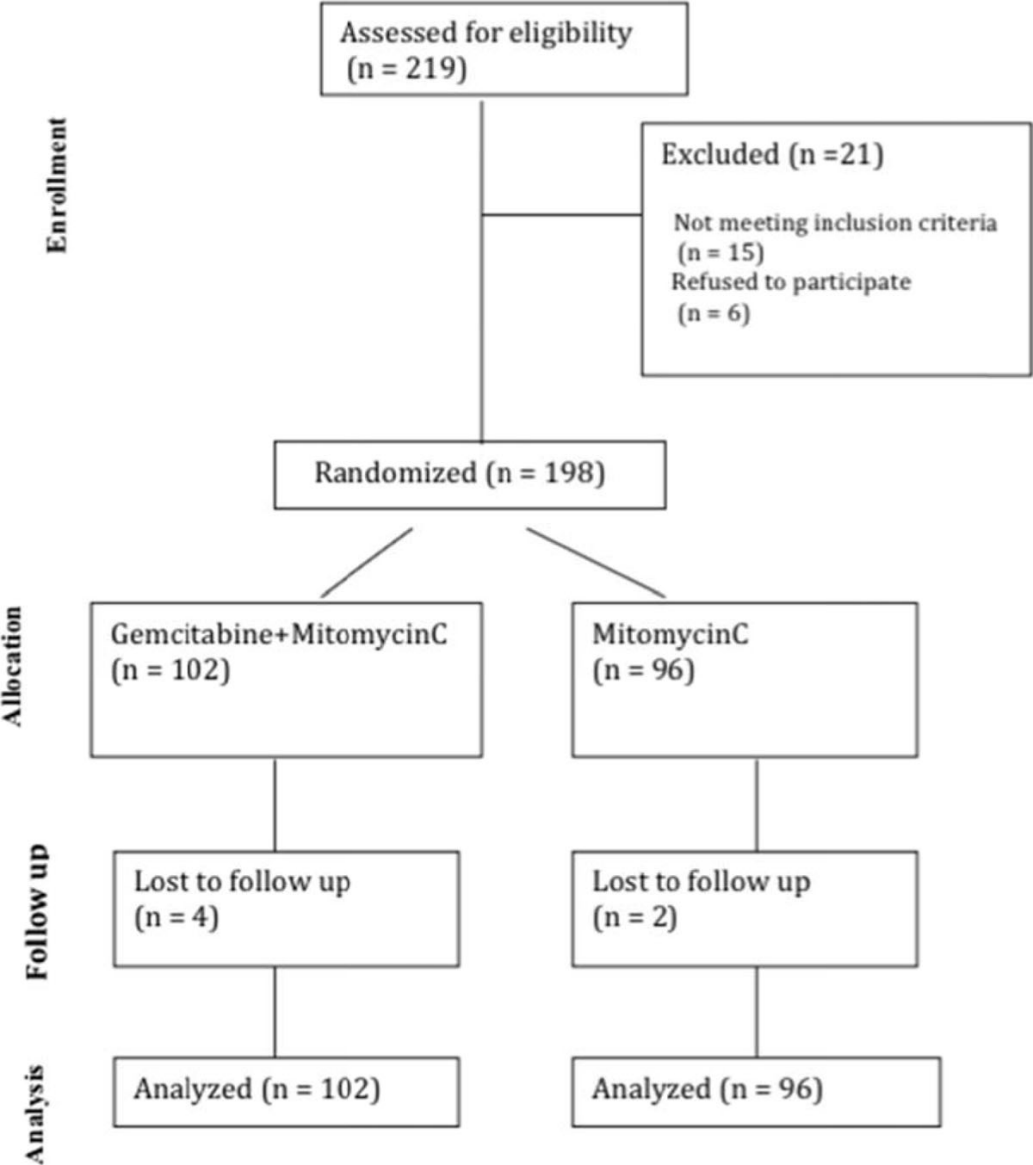
CONSORT diagram showing the participants through each stage of a randomized trial.

